# Proteomic Response to Acupuncture Treatment in Spontaneously Hypertensive Rats

**DOI:** 10.1371/journal.pone.0044216

**Published:** 2012-09-12

**Authors:** Xinsheng Lai, Jiayou Wang, Neel R. Nabar, Sanqiang Pan, Chunzhi Tang, Yong Huang, Mufeng Hao, Zhonghua Yang, Chunmei Ma, Jin Zhang, Helen Chew, Zhenquan He, Junjun Yang, Baogui Su, Jian Zhang, Jun Liang, Kevin B. Sneed, Shu-Feng Zhou

**Affiliations:** 1 Department of Acupuncture and Moxibustion, School of Acupuncture and Moxibustion, Guangzhou University of Chinese Medicine, Guangzhou, China; 2 Department of Human Anatomy, School of Fundamental Medical Sciences, Guangzhou University of Chinese Medicine, Guangzhou, China; 3 Department of Pharmaceutical Sciences, College of Pharmacy, University of South Florida, Tampa, Florida, United States of America; 4 Department of Human Anatomy, School of Medicine, Jinan University, Guangzhou, China; 5 Department of Acupuncture and Moxibustion, School of Chinese Medicine, Southern Medical University, Guangzhou, China; 6 Department of Surgery, The Third Hospital of Nanchang, Nanchang, Jiangxi, China; 7 Department of Pharmacotherapeutics and Clinical Research, College of Pharmacy, University of South Florida, Tampa, Florida, United States of America; Pennington Biomedical Research Center, United States of America

## Abstract

Previous animal and clinical studies have shown that acupuncture is an effective alternative treatment in the management of hypertension, but the mechanism is unclear. This study investigated the proteomic response in the nervous system to treatment at the Taichong (LR3) acupoint in spontaneously hypertensive rats (SHRs). Unanesthetized rats were subject to 5-min daily acupuncture treatment for 7 days. Blood pressure was monitored over 7 days. After euthanasia on the 7^th^ day, rat medullas were dissected, homogenized, and subject to 2D gel electrophoresis and MALDI-TOF analysis. The results indicate that blood pressure stabilized after the 5th day of acupuncture, and compared with non-acupoint treatment, Taichong-acupunctured rat’s systolic pressure was reduced significantly (*P*<0.01), though not enough to bring blood pressure down to normal levels. The different treatment groups also showed differential protein expression: the 2D images revealed 571±15 proteins in normal SD rats’ medulla, 576±31 proteins in SHR’s medulla, 597±44 proteins in medulla of SHR after acupuncturing Taichong, and 616±18 proteins in medulla of SHR after acupuncturing non-acupoint. In the medulla of Taichong group, compared with non-acupoint group, seven proteins were down-regulated: heat shock protein-90, synapsin-1, pyruvate kinase isozyme, NAD-dependent deacetylase sirtuin-2, protein kinase C inhibitor protein 1, ubiquitin hydrolase isozyme L1, and myelin basic protein. Six proteins were up-regulated: glutamate dehydrogenase 1, aldehyde dehydrogenase 2, glutathione *S*-transferase M5, Rho GDP dissociation inhibitor 1, DJ-1 protein and superoxide dismutase. The altered expression of several proteins by acupuncture has been confirmed by ELISA, Western blot and qRT-PCR assays. The results indicate an increase in antioxidant enzymes in the medulla of the SHRs subject to acupuncture, which may provide partial explanation for the antihypertensive effect of acupuncture. Further studies are warranted to investigate the role of oxidative stress modulation by acupuncture in the treatment of hypertension.

## Introduction

Hypertension is a multifactorial condition characterized by systolic blood pressure (SBP) of ≥140 mmHg or diastolic blood pressure (DBP) of ≥90 mmHg [1], [2]. Currently, estimates for the number of people worldwide affected by hypertension exceed 1 billion [3], including at least 76.4 million American adults ≥20 years of age (i.e. about one out of three U.S. adults is affected based on data from the 2005–2006 National Health and Nutrition Examination Survey) [4]. The prevalence of hypertension is almost the same between men and women in America. In 2010, hypertension raised a cost of $76.6 billion in health care services, medications, and missed days of work in the United States [5]. Despite the recent increase in public awareness of hypertension, only 78% of hypertensive patients were aware of their condition; 68% were using antihypertensive drugs; and 64% of those treated had their blood pressure controlled [4]. It has been projected that an additional 27 million American people could suffer from hypertension, a 9.9% increase in prevalence from 2010 [6]. Chronic hypertension is a major causative factor of morbidity and mortality, as uncontrolled hypertension can contribute to myocardial infarction, stroke, congestive heart failure, and renal failure. The overall death rate resulting from hypertension was 18.3 in the United States in 2008 [4]. Major barriers to successful conventional pharmacological treatment include side effects, out-of-pocket expenses, noncompliance of patients and improper dosage/regimen.

Acupuncture provides an alternative treatment approach to hypertension and has been a critical constituent of traditional Chinese Medicine (TCM) for the past 2,500 years [7], [8]. More recently, the practice of acupuncture has become prevalent in the United States, with over 2 million Americans reporting recent use of acupuncture [9]. In TCM, acupuncture theory is based on the premise that energy (called “Qi”) goes along determined pathways or meridians within the body and is responsible for maintaining good health by providing homeostatic regulation of vital body function [10]. In TCM, diseases are believed to result from imbalances and disturbances in the flow of “Qi” within the body, thus acupuncture consists of treatments by insertion and manipulation of needles at specific anatomic locations (acupoints) in the body with the intent of regulating the energy flow and restoring that balance. In acupuncture, the placement of needles into the body is dictated by the location of meridians, thought to mark patterns of energy flow throughout the human body in TCM [10]. Acupuncture has been widely used to manage musculokeletal pains, nausea secondary to surgery and chemotherapy, and other diseases.

A number of animal and clinical studies have reported the efficacy of acupuncture in reducing hypertension [11], [12], [13], [14]. Although there is some discrepancy between the reports, the majority of them indicate that acupuncture of traditional acupoints causes a significant decrease in blood pressure, while acupuncture at sham points does not [11]. However, the long-term effect and elucidation of the mechanisms through which acupuncture lowers blood pressure has not been reported. On a systemic level, studies have suggested the involvement of plasma renin, aldosterone, and angiotensin II activity [15], [16], [17], [18]. The involvement of increased sodium excretion [19], as well as changes in plasma norepinephrine, serotonin, and endorphin levels have also been implicated [20], [21], [22].

Although many studies have looked at the systemic effect of acupuncture on hypertension, less research has been conducted on the response to acupuncture at proteomic and cellular/subcellular levels. We hypothesize that acupuncture at proper acupoints will result in differential protein expression compared to acupuncture at sham points. Additionally, as hypertension is a multifactorial condition and acupuncture results in a multitude of *in vivo* changes, we believe that acupuncture will lower blood pressure through modulation of multiple biochemical and molecular pathways. This study is the first to examine the proteomic response in the medulla of rats to acupuncture at the LR3 point compared to stimulation at a sham-acupuncture site in spontaneously hypertensive rats (SHRs). Now rats are a well accepted animal model for acupuncture studies because both rats and humans share a number of anatomical and genomic features. A large number of published acupuncture studies use rats as the experimental model [23], [24], [25].

## Materials and Methods

### Ethics Statement

All animal experiments were conducted at the Laboratory Animal Center of Guangzhou University of Chinese Medicine, Guangzhou, China. The procedure was approved by the Ethics Committee of Guangzhou University of Chinese Medicine, Guangzhou, China [permit No.: SYXK (Yue) 2008-0085].

### Chemicals and Reagents

Chemicals and materials used for our proteomic study, including urea, CHAPS detergent, dithiothreitol (DTT), 2-mercaptoethanol, bio-lyte3/10, bromophenol blue, mineral oil, filter paper wicks, immobilized pH gradient (IPG) ready strip (11 cm, pH 5–8), 12.5% Tris-HCl, 1.5 M Tris-HCl (pH 8.8), iodoacetamide, ready prep overlay agarose, 10 × Tris/glycine/SDS buffer, precision plus unstained standard, and SYPRO^®^ Ruby protein gel stain were purchased from Bio-Rad Co. (Hercules, CA). Thiouea, protease inhibitor cocktail and a matrix solution of α-cyano-4-hydroxycinnamic acid in acetonitrile/methanol were purchased from Sigma-Aldrich Inc. (St Louis, MO). For matrix-assisted laser desorption/ionization time-of-flight (MALDI-TOF) mass spectrometry (MS) experiments, pure trypsinogen was obtained from Shanghai Sangon Biotech Co. Ltd (Shanghai, China). For enzyme immunoassay experiments, reagent kits for measuring rat glutathione *S*-transferase M5 (GSTM5), aldehyde dehydrogenase 2 (ALDH2), and protein kinase C (PKC) were purchased from R&D Systems Inc. (Minneapolis, MN).

### Animals

Due to supply shortage in China, we could not get Wistar Kyoto rats during our experimental period. Based on literature reports, Sprague Dawley (SD) rats have been used as the normotensive controls for spontaneously hypertensive rats [26], [27]. In fact, SD rats are originally derived from Wistar Kyoto rats with minor physiological and biochemical differences.

A total of 54 9-week-old male SHRs weighing 180–200 g and body weight-matched 18 SD rats were obtained from Beijing Vital River Laboratory Animals Co. Ltd (Beijing, China). They were housed at a controlled ambient temperature of 22–25°C with 55±5% relative humidity and a 12 hr light/12 hr dark cycle (lights on at 8:00 AM) at the Laboratory Animal Center of Guangzhou University of Chinese Medicine, Guangzhou, China. The animals were given food and water *ad libitum* for 3 days, and were acclimatized to handling by the researchers and BP-measuring conditions for 1 week prior to acupuncture treatments. After measuring BP, the SHRs (BP≥140 mmHg) were randomly divided into three groups: the Taichong group (n = 18) was treated with acupuncture at the LR3 acupoint, the non-acupoint group (n = 18) was treated at non-acupoints, and the model group (n = 18) was untreated throughout the duration of the experiment. Body weight-matched SD rats with normal BP (normal group) were used as comparative control and were untreated throughout the duration of the experiment (n = 18).

### Acupuncture Treatment

**Figure 1:**
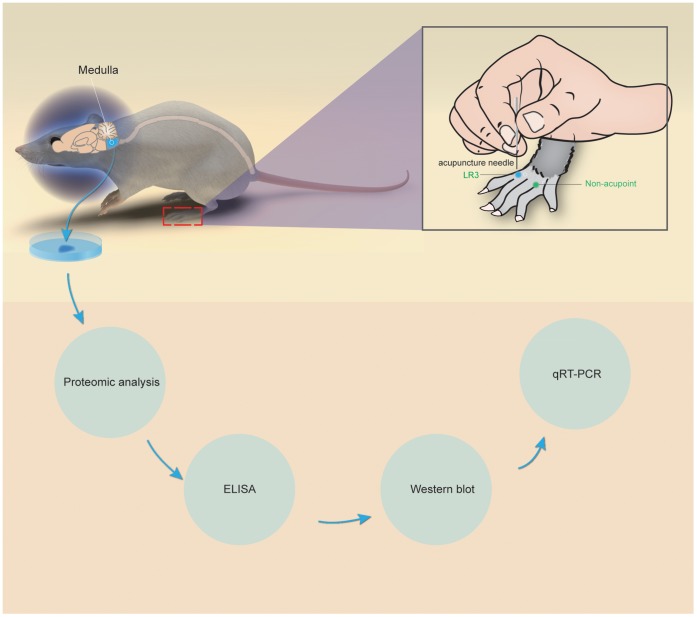
The Taichong (LR3) point and non-acupoint in hypertensive rats.

In one group of the SHRs, acupuncture was performed at bilateral Taichong points (LR3) located between the 1^st^ and the 2^nd^ metatarsal of dorsal foot; while in non-acupoint group acupuncture was done at bilateral non-acupoint located at the fossa between the 3^rd^ and 4^th^ metatarsal of dorsal foot (Figure 1). The locations for sham vs nonsham acupoints are based on the anatomic locations in TCM that have been mapped to rats by other researchers [28], [29], [30]. Two researchers, a technician and a trained acupuncturist (Dr Jiayou Wang), completed the acupuncture procedure in Taichong group and non-acupoint groups. The acupuncture procedure was performed on a heated table; and the technician and acupuncturist were seated across from one another. Each unanesthetized rat was placed headfirst into a homemade black restraint cone (similar to the pastry bags used by bakers) so that the anterior portion rats’ body was firmly in the cone, while the posterior portion was exposed. A zip tie was used to secure the restraint cone around the rats’ bodies, while the technician fixed the rats’ hind legs in place. The acupuncturist then bi-laterally inserted the acupuncture needle 3 mm deep into the appropriate location. The needle was twisted 180 degrees at a rate of 80±5 times per min. Acupuncture treatment was given daily (5 min per treatment) for 7 days. The acupuncture procedure was carried out with extremely gentle operation to avoid any unnecessary stimulus and stress to the rats.

### Measurement of Blood Pressure

Blood pressure measurements were performed by two experienced technicians directly after acupuncture. SBP was measured non-invasively by the tailcuff method after at least a 5-min resting period using the BP-6A blood pressure measuring system from Chengdu TME Technology Co. Ltd (Chengdu, China). Immediately after acupuncture treatment, each rat was gently placed into restraint cones and their tails were fixed using the rat-tail fixing facility. The ventral portion of each rat was placed on the heat pad, while the BP measurement cuffs were put in place. Once a batch of 6 rats was in place, SBP was recorded by the pulse recording sensor facility following a 5-min warm-up period. Ten preliminary cycles (10×1.5 min) were performed to allow the rats to adapt to the rat-tail fixing facility. After the preliminary cycles, 5 cycles were recorded at each time point without intervention. To ensure accuracy and reproducibility, the rats were trained for 1 week prior to the experiment, and measurements were taken at the same time each day. During experiments, all animals were handled with extreme caution to minimize stress to the rats. Two BP-6A measuring systems were used so that blood pressure measurements could be done concurrently with acupuncture.

### Tissue Preparation

The animals were anesthetized with an overdose of sodium phenobarbital (50 mg/kg body weight) by intraperitoneal injection on the 7^th^ day after starting the acupuncture treatment and perfused intracardially with 50 ml physiological saline. The brain capsule was removed carefully and the medulla quickly dissected and collected. The medulla was preserved using liquid nitrogen until analysis. Twelve medullas (three medullas per group) were used to run the 2D gel assay. Each medulla was added in 700 ml buffer containing 7 mol/L urea, 2 mol/L thiourea, 2% CHAPS, 1% Triton X-100, 1% cocktail, and ultrasonicated for 5 sec three times. The mixture was centrifuged at 1,500 *g* for 10 min, and the supernatants were collected.

### Two Dimensional (2D) Gel Running

A tube gel running system was used for first-dimensional running with 100 mmol/L sodium hydroxide, cathode buffer, 10 mmol/L phosphoric acid, and anode buffer. Pre-cast carrier ampholyte tube gels (pH 5–8, 11 cm) were prefocused with a maximum of 1,500 V and 110 µA per tube. The protein samples of 160 µg were loaded into the tube gels and focused for 17 hr and 30 min to reach 18,000 Vh.

The gels were extruded from the tubes after completion of focusing and were incubated in premixed Tris acetate equilibration buffer with 0.01% bromphenol blue and 50 mmol/L dithiothreitol for 2 min before loading onto pre-cast 50 mmol/L dithiothreitol for 2 min before loading onto pre-cast 10% homogeneous, 200×200 mm slab gels. The upper running buffer contained 0.2 mol/L Tris base and 0.2 mol/L Tricine. The system was run with a maximum of 500 V and 20,000 mW per gel.

The gel slabs were fixed in 10% methanol and 7% acetic acid for 30 min. The fixed solution was removed, and 500 ml of SYPRO^®^ Ruby gel stain was added to each gel and incubated on a gently continuous rocker at room temperature for 16 hr.

### Image Analysis

The gel images were obtained with Typhoon9200 scanner (GE Healthcare Co., Piscataway, NJ). Imagesaster 6.0 2D software was used for matching and quantitative analysis of the protein spots on the gels. The average gel was constructed as a representative gel for the three medulla samples taken from each group of rats. The average mode of background subtraction was used for normalization of intensity volume that represents protein concentration or amount on each spot. The average gel was then used for determination of the existence of difference of protein expression levels between each group.

### Matrix Assisted Laser Desorption/ionization Time-of-flight (MALDI-TOF) and Data Analysis

Tryptic digests were analyzed using 2 separate instruments, an electrospray Q-TOF-2 mass spectrometer coupled with capillary high-performance liquid chromatography and MALDI Ultraflex TOF-TOF (Bruker Daltonics Inc., Fremont, CA). Protein identification from the tandem mass spectrometry (MS/MS) data was done by searching the National Center for Biotechnology Information nonredundant database with the GPS software, to search and identify proteins in the MASCOT database.

### Quantitative Reverse Transcription-polymerase Chain Reaction (qRT-PCR)

Twenty four medullas (six medullas from each group) were used to run the qRT-PCR. The medulla was homogenized in 400 µL of TRIzol reagent (Invitrogen, Grand Island, NY). To avoid contamination with genomic DNA, the RNA samples were treated with RNase-free DNase (Promega, Madison, WI). Reverse transcription was performed using M-MLV reverse transcriptase (Promega, Madison, WI).

The 18srRNA gene (112 bp, F: 5′-CCTGGATACCGCAGCTAGGA; R: 5′-GCGGCGCAATACGAATGCCCC), synapsin I gene (160 bp, F: 5′-ATGGGCAAGGTCAAGGTAGA; R: 5′-ATGTCCTCATGTAGGCCTTGT) and myelin basic protein genes (152 bp, F: 5′-AACGCAGGGACGAAACTT; R: 5′-CCAAGAACAGTAGGTGCTTCT ) were tested in this study. Real-time RT-PCRs were carried out using an ABI PRISM® 7500 Sequence Detection System (Applied Biosystems, Carlsbad, CA) and the SYBR Green PCR Master Mix kit (Toyobo Co. Ltd., Osaka, Japan). The expression of the target genes was assessed in relation to a housekeeping gene (18 srRNA) using the comparative (2-ΔΔCT) method in each sample. Fold differences against average values of SD rats were calculated.

### Western Blotting Assay

Twelve medullas (three medullas from each group) were used to run the Western blotting assay. The medulla was homogenized in the lysis buffer (0.05 mol/L Tris-HCl at pH 7.4, 0.15 mol/L NaCl, 0.001 mol/L EDTA, 0.001 mol/L EGTA,1% Triton X-100, and 1% cocktail) using an ultrasound homogenizer at 50 Hz. The lysate was then centrifuged at 12, 000×*g* for 10 min. The protein concentration of the supernatant was measured with the Bradford protein assay. The supernatant was heated in the 5 × SDS sample buffer at 95°C for 10 min. An aliquot of 30 µg of the sample was loaded into a 10% polyacrylanide gel and separated at 120 V. Subsequently, proteins on the gel were transferred to PVDF membrane. The membrane was incubated with synapsin-1 antibody (1∶1,000, Sigma-Aldrich, St Louis, MO), or myelin basic protein antibody (1∶1,200, Sigma-Aldrich, St Louis, MO) overnight at 4°C, rinsed with TBST buffer, and then incubated with anti-rabbit IgG (1∶5,000 Dako, Glostrup, Denmark) or anti-mouse IgG (1∶4,000 Dako, Glostrup, Denmark) for 1 hr at room temperature. The immune complexes were detected by ECL and exposed to X-ray film.

### Enzyme-linked Immunosorbent Assay (ELISA)

Twenty four (six medullas from each group) were used to carry out the ELISA. The medulla was ultrasonicated in PBS (pH7.4) and centrifuged at 1,000×*g* for 25 min; the supernatants were collected. The tissues were manipulated according to the instructions of the commercially available kits.

### Statistical Analysis

Data analysis was conducted by either t test or one-way analysis of variance (ANOVA). Statistical significance was considered only when *P*<0.05.

## Results

### Effect of Acupuncture on Systolic Pressure in SHRs

**Figure 2:**
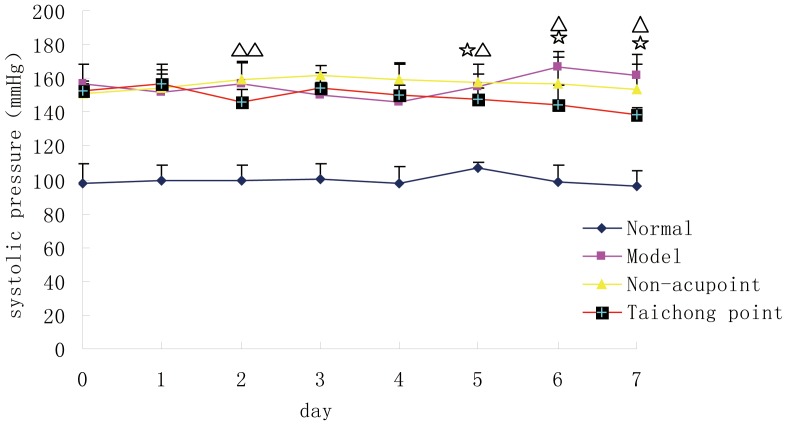
Effect of acupuncture on systolic pressure in 4 groups of rats. ^△△^ *P*<0.01, the 1^st^ vs. 2^nd^ day in Taichong group;^☆△^ *P*<0.01, Taichong group vs. non-acupoint, the 1^st^ vs. 5^th^ day in Taichong group; ^△^ *P*<0.01, Taichong group vs. model group, the 1^st^ vs. 6^th^ or 7th day in Taichong group; ^☆^ *P*<0.01, the 4^th^ vs. 6^th^ or 7th day in model group.

Due to the acclimation period, the rats did not experience excess stress during the acupuncture treatment. Before acupuncture, the SBP of Taichong group, and the non-acupoint group and model group were insignificantly different (*P*>0.05), but significantly higher than the normal rats. On the 1st day after starting the acupuncture treatment, the SBP between the Taichong group, the non-acupoint group and model group were insignificantly different (*P*>0.05). On the 2nd day after acupuncture, compared with the model group and non-acupoint group, the SBP of Taichong group was significantly decreased (*P*<0.01, Figure 2). On the 3rd day after acupuncture, compared with Taichong group, the SBP of the non-acupoint group was significantly higher, suggesting that the acupuncture at non-acupoint may serve as a stimulus, resulting in a significantly increased SBP in rats. On the 4th day after acupuncture, the SBP among Taichong, non-acupoint and model groups were insignificantly different (*P*>0.05). On the 5th day of acupuncture, compared with non-acupoint, Taichong group’s SBP reduced significantly (*P*<0.01). On the 6^th^ or 7th day after acupuncture, compared with the model group and non-acupoint group, Taichong group’s SBP were significantly lower (*P*<0.01, Figure 2), suggesting acupuncture at Taichong points begin to stabilize the blood pressure since the 5^th^ day after acupuncture. In the model, Taichong and non-acupoint groups, the SBP values were significantly higher than the normal group (*P*<0.01) over the experiment period, suggested that acupuncture at Taichong or non-acupoint were unable to reduce the systolic pressure to normal levels in this study.

The results of between-day comparison in each group are as follows: compared with the 1st day, the SBP of Taichong group decreased significantly on the 2nd day (*P*<0.01); but on the 3rd and 4th day, the SBP was increased again (*P*>0.05), and then began to decrease at the 5th day (*P*<0.01), until the 6th and 7th day, at which point the level of SBP remained stable (*P*<0.01, Figure 2), suggesting that acupuncture at Taichong point begin to stabilize the blood pressure level since the 5^th^ day. Non-acupoint group’s SBP were insignificantly altered in any time period (*P*>0.05). Compared with the 3rd day, the model rats’ SBP was significantly increased on the 6th day (*P*<0.01); compared with the 4th day, model group’s SBP was also significantly increased on the 6th day and the 7th days (*P*<0.01), suggesting that SBP of model group was rising. Rats in the normal group had stable and normal SBP over the experiment period (*P*>0.05).

### Differential Protein Expression Profiles in Different Groups of Rats

**Figure 3:**
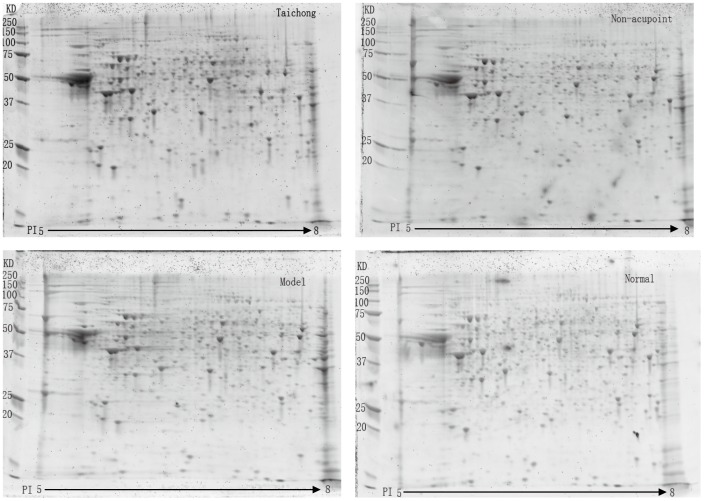
Protein profiles of rat medulla obtained over different pI ranges.

**Figure 4:**
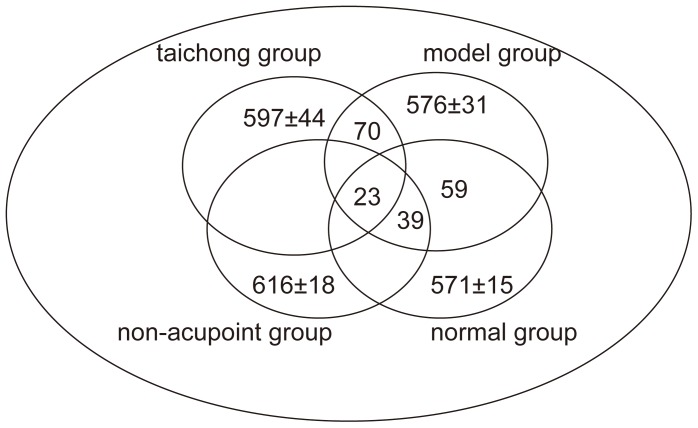
Venn diagrams. A, the differentially expressed protein spots by four groups; and B. the identified proteins by 2D gel ± MALDI-TOP MS/MS.

The 2-DE image of rat medulla in each group is shown in Figure 3. In this study, 2-DE images revealed 571±15 proteins in normal SD rats’ medulla, 576±31 proteins in SHR’s medulla, 597±44 proteins in the medulla of SHRs after acupuncturing Taichong, and 616±18 proteins in the medulla of SHRs after acupuncturing non-acupoint (for Venn diagrams, see Figure 4). Compared with model group, 70±15 proteins were found differentially expressed in the medulla from Taichong group (expression ratio of protein was >2 folds, Table 1); 39±7 proteins were found differentially expressed in medulla oblongata from non-acupoint group (expression ratio of protein was >2 fold); and 59±12 proteins were found differentially expressed in medulla oblongata from normal group (expression ratio of protein was >2 fold). However, after artificial comparative analysis, it was found that there were only 23 protein spots all showing differential expression on 2-D gels among 4 groups.

**Table 1:** Number of differentially expressed proteins compared to the model group.

Group	No. of protein spots identified by image 2D software (between 2 groups)	No. of protein spots identified by artificial comparative analysis (among 4 groups)	No. of proteins identified by MALDI-TOP MS/MS
Normal	59±12	23	22
Taichong	70±15	23	22
Non-acupoint	39±7	23	22

### Identification of Differentially Expressed Proteins in Different Groups of Rats

In this study, 23 protein spots were cut and proteins identified from 2D gel and MALDI-TOP MS (Figure 4). Among these spots, 22 proteins were identified by MALDI-TOP MS, with 14 of which being functional proteins and further analyzed (Table 2 & Table 3). The latter group of proteins included spots 69, 148, 273, 274, 306, 485, 497, 571, 603, 605–1, 605–2, 683, 739, and 754.

**Table 2:** Differentially expressed proteins among 4 groups of rats.

Spot num.	Swiss-Prot Accession NO.	Protein name	Protein Score C.I.%/Total Ion Score C.I.%	Sequence Coverage %	Major function
1	IPI00231023	Synapsin-1	100/100	28	Adjust release of neurotransmitters
2	IPI00607210	MBP	100/100	90	Form medulla sheath
3	IPI00324893	KCIP-1	100/100	55	Signal transduction
4	IPI00205332	α-ETF	100/100	51	Regulate oxidative stress
5	IPI00562798	SIRT2	100/100	31	Oxidative phosphorylation
6	IPI00324633	GLUD1	100/100	44	Amino acid oxidation; chaperones
7	IPI00197770	ALDH2	100/100	31	Regulate oxidizing reaction
8	IPI00208636	GSTM5	100/100	31	Anti-oxidative stress
9	IPI00231643	SOD	100/100	52	Anti-oxidative stress
10	IPI00196994	ARHGDIA	100/100	56	Revascularization
11	IPI00212523	DJ-1	100/100	56	Oxidative stress
12	IPI00210566	HSP90-α	100/100	34	Amino acid oxidation; chaperones
13	IPI00231929	Pyruvate kinase isozyme	100/100	40	Protein catabolism
14	IPI00204375	UCHL1	100/100	51	Protein catabolism

**Table 3:** The expression of protein spots in the medulla of four different groups of rats.

Spot	Protein	% Volume (mean ± SD)			
		Model	Taichong point	Non-acupoint	Normal
1	Synapsin-1	0.574±0.004*	0.119±0.007#	0.673±0.006	0.179±0.003^▪^
2	MBP	1.24±0.036*	0.119±0.033^#▾^	1.32±0.033	0.683±0.127^▪^
3	KCIP-1	0.747±0.038*	0.148±0.012#	0.665±0.059	0.116±0.001^▪^
4	α-ETF	0.044±0.005*	0.014±0.004	0.014±0.004▴	0.023±0.005
5	SIRT2	0.275±0.011*	0.086±0.011#	0.27±0.021	0.09±0.001^▪^
6	GLUD1	0.021±0.001*	0.037±0.004#	0.022±0.002	0.039±0.001^▪^
7	ALDH2	0.08±0.007*	0.180±0.005#	0.084±0.006	0.187±0.006^▪^
8	GSTM5	0.109±0.011*	0.155±0.003#	0.107±0.018	0.147±0.008^▪^
9	SOD	0.074±0.012*	0.223±0.017^#▾^	0.077±0.008	0.169±0.006^▪^
10	ARHGDIA	0.122±0.005*	0.242±0.025^#▾^	0.025±0.004	0.024±0.02
11	DJ-1	0.046±0.004	0.283±0.031^#▾^	0.124±0.029▴	0.031±0.009^▪^
12	HSP90-α	0.571±0.056	0.637±0.046▾	0.776±0.051▴	0.226±0.019^▪^
13	Pyruvate kinase isozyme	0.185±0.029*	0.067±0.017#	0.145±0.045	0.076±0.019
14	UCHL1	0.148±0.007	0.156±0.006^#▾^	0.37±0.051▴	0.354±0.019

* *P*<0.01, model group vs. Taichong point group or normal group;

# *P*<0.01, Taichong group vs. non-acupoint group;

▾ *P*<0.01, Taichong group vs. normal group;

▴ *P*<0.01, non-acupoint group vs. model group; and ^▪^ *P*<0.01, non-acupoint group vs. normal group.

In the medulla of the Taichong group, compared with model group, six proteins were significantly down-regulated: synapsin-1, myelin basic protein (MBP), pyruvate kinase isozyme, protein kinase C inhibitor protein 1 (KCIP-1), electron transfer flavinprotein subunit-α (α-ETF), and NAD-dependent deacetylase sirtuin-2 (SIRT2). Six proteins were up-regulated: glutamate dehydrogenase 1 (GLUD1), ALDH2, GSTM5, superoxide dismutase (SOD), Rho GDP dissociation inhibitor 1 (ARHGDIA), and DJ-1 protein (Table 4).

**Table 4:** Relative protein expression among four different groups of rats.

Protein	Up/Down-regulation			
	Model vs. Normal	Taichong vs. Model	Taichong vs. Non-Acupoint	Non-Acupoint vs. Model
**Oxidative Stress**				
α-EFT	**↑**	**↓**	**⊤**	**↓**
HSP90-α	**↑**	**⊤**	**↓**	**↑**
SOD	**↓**	**↑**	**↑**	**⊤**
DJ-1	_Τ_	**↑**	**↑**	**↑**
GSTM5	**↓**	**↑**	**↑**	**⊤**
ALDH2	**↓**	**↑**	**↑**	**⊤**
GLUD1	**↓**	**↑**	**↑**	**⊤**
**Neurotransmitter Release**				
Synapsin-1	**↑**	**↓**	**↓**	**⊤**
**Revascularization**				
ARHGDIA	**⊤**	**↑**	**↑**	**⊤**
**Protein Catabolism**				
Pyruvate kinase isozyme	**↑**	**↓**	**↓**	**⊤**
UCHL1	**↓**	**⊤**	**↓**	**↑**
**Oxidative Phosphorylation**				
SIRT2	**↑**	**↓**	**↓**	**⊤**
**Medullary Sheath**				
MBP	**↑**	**↓**	**↓**	**⊤**
**Signal Transduction**				
KCIP-1	**↑**	**↓**	**↓**	_⊤_

**↑: up-regulated; ↓: down-regulated; Τ: unchanged.**

In the medulla of non-acupoint group, compared with model group, only one protein was down-regulated: electron transfer flavoprotein; but three proteins were up-regulated: heat shock protein 90 (HSP90), ubiquitin hydrolase isozyme L1 (UCHL1) and DJ-1 protein (Table 4).

In the medulla of Taichong group, compared with non-acupoint group, seven proteins were down-regulated: HSP90, synapsin-1, pyruvate kinase isozyme, SIRT2, KCIP-1, UCHL1, and MBP. Six proteins were up-regulated: GLUD1, ALDH2, GSTM5, ARHGDIA, DJ-1 protein and SOD.

In the medulla of model group, compared with normal group, five proteins were down-regulated: GLUD1, GSTM5, ALDH2, UCHL1 and SOD. Seven proteins were up-regulated: HSP90, synapsin-1, pyruvate kinase isozymes, SIRT2, α-EFT, KCIP-1, and MBP (Table 4).

In the medulla of Taichong group, compared with normal group, two proteins were down-regulated: UCHL1 and MBP. Four proteins were up-regulated: HSP90, ARHGDIA, DJ-1 protein and SOD.

In the medulla of non-acupoint group, compared with the normal group, four proteins were down -regulated: GLUD1, ALDH2, GSTM5 and SOD; six proteins were up-regulated: HSP90, synapsin-1, SIRT2, KCIP-1, DJ-1 and MBP (Table 4).

### Data from qRT-PCR, ELISA and Western Blotting Assays

To further verify the reliability of the proteomic analysis, the expression of synapsin I and MBP at protein and mRNA levels were analyzed by Western blot and qRT-PCR, respectively. In addition, the GSTM5, ALDH2 and PKC levels in medullas were determined by ELISA.

**Figure 5:**
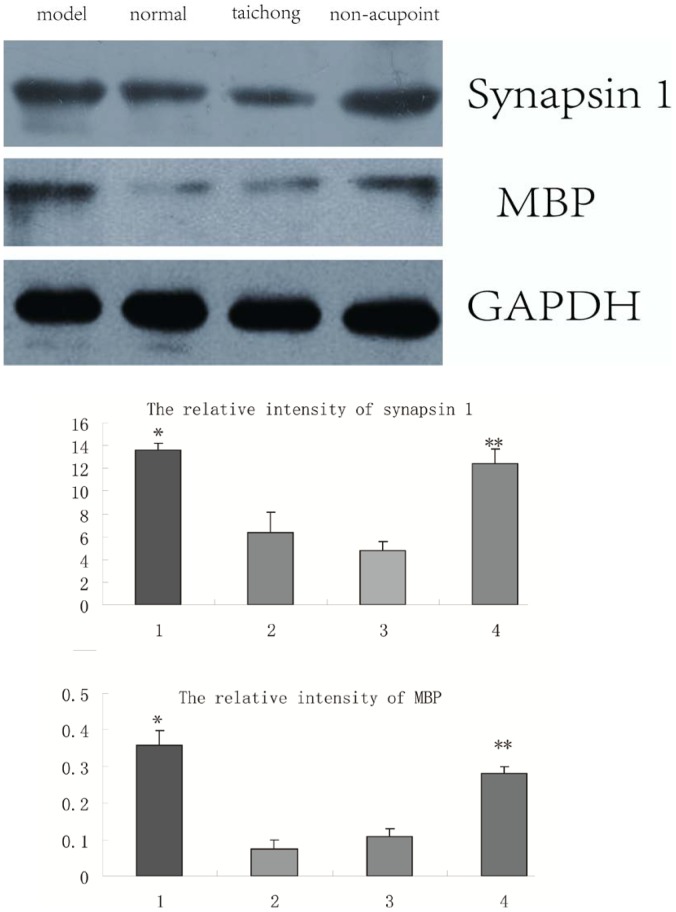
Blots of Western blot assays for synapsin-1 and myelin basic protein (MBP) in the medulla of SHRs. *P<0.05; **P<0.01.

Our Western blot analysis revealed that synapsin I was significantly increased in SHRs compared to age-matched normotensive SD rats, and remained unchanged after acupuncture treatment at non-acupoint but was down-regulated after acupuncture treatment at Taichong point (Figure 5). MBP was also significantly increased in 9-week-old SHRs compared to age-matched normotensive SD rats, and remained unchanged after acupuncture treatment at non-acupoint but was down-regulated after acupuncture treatment at Taichong point.

**Figure 6:**
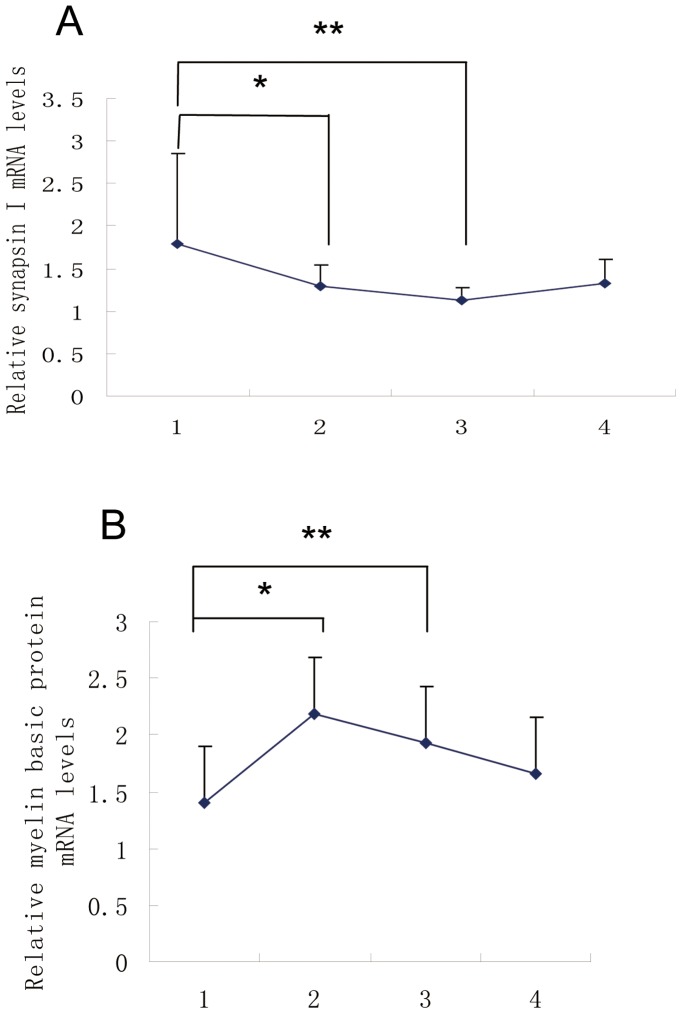
Blots of qRT-PCR assays for the mRNA levels of synapsin-1 and myelin basic protein (MBP) genes in the medulla of SHRs. Lane 1, model group; lane 2, normal group; lane 3, Taichong group; and lane 4, non-acupoint group. *P<0.05; **P<0.01.

Our qRT-PCR assay revealed that synapsin I was up-regulated in 9-week-old SHRs compared to age-matched normotensive SD rats, and remained unchanged after acupuncture treatment at non-acupoint but were down-regulated after acupuncture treatment at Taichong point (Figure 6). The mRNA of MBP was down-regulated in SHRs compared to age-matched normotensive SD rats, and remained unchanged after acupuncture treatment at non-acupoint but were up-regulated after acupuncture treatment at Taichong point.

The results of synapsin I by Western blot and qRT-PCR assays were consistent with those from 2D gel analysis. Although the results of MBP by Western blot assay were consistent with those by 2-D gel analysis, but were opposite to those from qRT-PCR.

**Figure 7:**
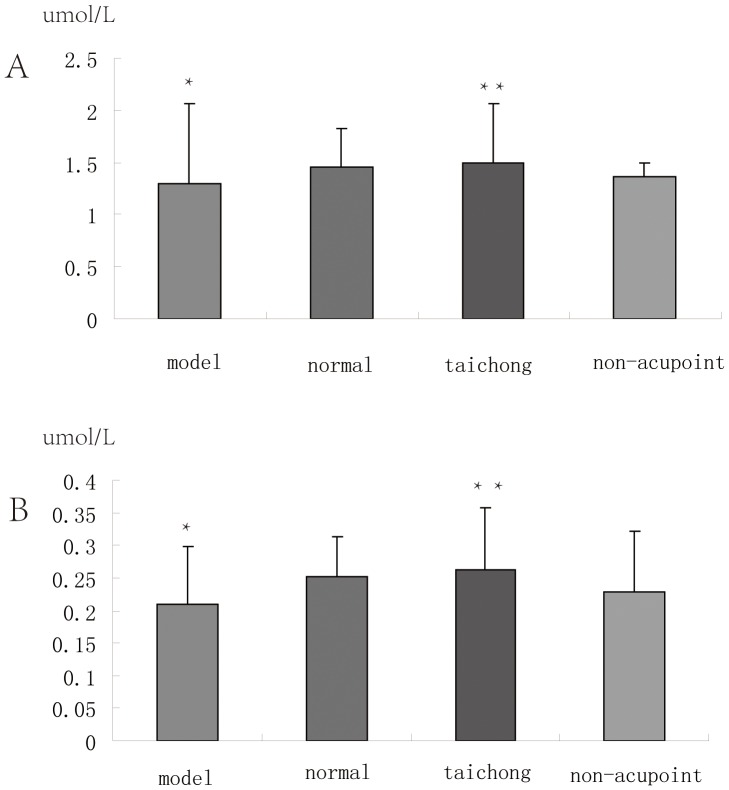
Content of glutathione *S*-transferase M5 and aldehyde dehydrogenase 2 in rat medulla by ELISA.

Our ELISA results of GSTM5 and ALDH2 are consistent with the results of 2D gel (Figure 7). In the medulla of model group, compared with normal group, the expression of GSTM5 was significantly down-regulated (*P*<0.01). After acupuncture treatment, the expression of GSTM5 in the medulla of Taichong group were significantly up-regulated (*P*<0.01); but the expression of GSTM5 in the medulla of non-acupoint group has no significant change compared to the model group (*P*>0.01), but lower than the normal group (*P*<0.01).

In the medulla model group, compared with normal group, the expression of ALDH2 was significantly down-regulated (*P*<0.01). After acupuncture treatment, the expression of ALDH2 in the medulla of Taichong group were significantly up-regulated (*P*<0.01), but the expression of ALDH2 in the medulla of non-acupoint group has no significant change compared to the model group (*P*>0.01), but lower than the normal group (*P*<0.01).

The expression of PKC in the medulla of model group has no significant change compared to the normal group (*P*>0.01). After acupuncture treatment, the expression of PKC in the medulla of Taichong group was the lowest compared to the model, normal, and non-acupoint group, but did not achieve statistical significance (*P*>0.01) (data not shown).

## Discussion

Although there has been some discordance as to the efficacy of acupuncture in hypertension [13], [15], [31], our results indicate that acupuncture at the Taichong (LR3) acupoint does alleviate high blood pressure due to essential hypertension, though this treatment alone is not able to bring blood pressure down to normal levels. When discussing the efficacy of acupuncture as a treatment for hypertension, it is important to distinguish between the short-term and long-term effect of acupuncture on hypertension. Our study, like most others, reports the short-term effect of acupuncture (directly following treatment) on blood pressure. Due to the proteomic nature of our study and the necessary euthanasia of the rats, the long-term effect of acupuncture on blood pressure regrettably could not be studied.

There are remarkable gender differences in many physiological parameters in health and disease. To avoid the interfering effect of female sex hormones on blood pressure, we have chosen male animals in this study. Studies have revealed sex hormones play a role in the regulation of blood pressure and development of hypertension [32]. The prevalence of hypertension is predicted to increase more among women than men. When rats were fed with an 8% NaCl diet, female rats became less hypertensive than male rats [32], which suggests that female sex hormones protect against the development of hypertension.

As stabilization of blood pressure in the Taichong group takes 5 days, the lag time between acupuncture treatment and stabilization of blood pressure suggests activation of specific metabolic pathways, causing changes at the protein expression level that contribute to the alteration at the cellular level. Changes at the cellular level eventually induce reduction in blood pressure at the systemic level. Analysis of the effects of acupuncture at the Taichong point in SHRs reveals a complex scenario of dramatic changes in abundance of various cellular proteins. Analysis of the fourteen statistically significant differentially proteins between the groups provided insights into the potential mechanisms through which acupuncture may reduce hypertension. As acupuncture is a general treatment with “pleiotropic” responses, the simultaneous activation of multiple therapeutic mechanisms is expected. The results are in accordance with our belief that multiple mechanisms play a role in alleviation of hypertension. Due to the complexity and underlying discord relating to the pathogenic factors associated with essential hypertension, this discussion will address the major changes in the proteomic/peptidomic analysis and how they may tie in with the incomplete reversal of hypertension pathogenesis in SHRs. The potential mechanisms involved include: reduction of oxidative stress, sympathetic modulation via synapsin-1, as well as NO level modulation.

As shown in Table 2 & Figure 3, protein expression in the model SHR rats is significantly different from those of normal (non-SHR) rats. The proteomic response that contributes to the SHR phenotype and reduction of hypertension in Taichong-needled rats includes the modulation of seven proteins related to oxidative stress, including SOD, ALDH2, GSTM5, GLUD1, protein DJ-1, HSP90α, and α-ETF. Many studies have reported the involvement of oxidative stress in hypertension, but questions have been raised as to whether oxidative stress causes hypertension, or if hypertension causes oxidative stress: a “chicken or egg” scenario. Oxidative stress results in the formation of reactive oxygen species (ROS), which are present in low levels in normal cells and functions and play an important role in vascular biology in regards to cell signaling and vascular contraction-relaxation. Delano et al. and other authors [33], [34], [35], [36] reported that oxidative stress might be a more global condition not only confined to vascular tissues, and more recently a number of reports have been published relating to the modulatory effect of ROS on hypertension in the brain.

Of these proteins, SOD, ALDH2, GSTM5, DJ-1 and GLUD1 were up-regulated, while electron α-ETF and HSP90α are down-regulated with Taichong treatment when compared to the model SHR rats. SOD, a protein involved in the dismutation of the superoxide anion into oxygen and hydrogen peroxide, is an important antioxidant defense in most cells. GSTM5 is involved in the conjugation of reduced glutathione to a wide number of exogenous and endogenous hydrophobic electrophiles, resulting in the detoxification of many oxidative stress proteins. GLUD1, primarily a mitochondrial matric enzyme, is involved in oxidative deamination and ammonia detoxification. ALDH2, another mitochondrial protein, functions to catalyze the oxidation of aldehydes and is also necessary for the bioactivation of organic nitrates, which are molecules with high vasodilator potency. The DJ-1 protein protects against oxidative stress and hydrogen peroxide based cell death, especially in neurons. HSP90α is a heat shock protein that promotes structural maintenance cell cycle control, and signal transduction, especially during stressful conditions; while α-ETF is a mitochondrial matrix protein that functions as a primary electron acceptor for primary dehydrogenase.

While the proteomic response of oxidative stress proteins demonstrates a complex, interweaved modulation, the predominant up-regulation of enzymes involved in ROS removal suggest an overall decrease in oxidative stress levels intracellularly due to acupuncture. The two oxidative stress related proteins down-regulated in response to acupuncture, HSP90α and α-ETF, play a major role when stressful conditions present, so reduction of oxidative stress should result in the down-regulation of these proteins. Furthermore, many other studies have linked acupuncture with a reduction in oxidative stress [37], [38]. ROS have many effects intracellularly as they function in signal transduction. This is the first report to implicate oxidative stress reduction as a possible mechanism of the therapeutic effect of acupuncture in hypertension. Acupuncture at the Taichong point changes the protein expression such that it reverses the changes seen in the proteomics analysis of SHR model compared to the normal rat, moving SHR rats back towards a normal protein expression as it related to hypertension. Additionally, it is interesting to note that multiple mitochondrial proteins have modulated protein levels after acupuncture treatment; additional research must be done to determine if mitochondrial function is in any way linked to the alleviation of hypertension due to acupuncture.

An up-regulation of oxidative stress enzymes and a decrease of oxidative stress may have multiple effects through which blood pressure is modulated. An increase in ROS in the rostral ventrolateral medulla (RVLM) contributes to the neural pathogenesis of hypertension [39]. In the RVLM, Nox-induced ROS initiate a forward loop in cross-activation of different receptors and between Nox and mitochondrial ROS [40]. Thus, a decrease in ROS due to acupuncture may oppose this pathogenic mechanism of hypertension. Additionally, the caudal ventrolateral medulla (CVLM) provides inhibitory input to the RVLM of the brain. A lack of these inhibitory inputs is believed to in part play a role in the neuropathogenesis of hypertension [41]. The increase in oxidative stress seen in hypertension could affect the frequency of these inhibitory inputs, thus the reduction of oxidative stress seen in acupuncture may provide a mechanism through with CVLM inhibitory input to the RVLM is augmented. In addition, paraventricular nucleus (PVN) of the hypothalamus plays a major role in autonomic and neuroendocrine regulation of blood pressure and body fluid homeostasis [42]. Functional studies have demonstrated the involvement of PVN in the control of fluid electrolyte homeostasis, feeding behavior, cardiovascular regulation, and stress adaptation [42]. It is unclear whether acupuncture affects the regulatory function of these regions through modulation of ROS.

It is technically difficult to oblate specific nuclei from the rat medulla and to collect enough samples for our proteomic and biochemical analysis. Since multiple regions/nuclei of the medulla are involved in blood pressure regulation, we collected the whole medulla for our study. To examine whether the proteins located in specific nuclei of the medulla are related to blood pressure modification by acupuncture, we have conducted a preliminary immunohistochemical study and shown altered MBP and synapsin I expression in some specific regions (e.g. NTS and RVLM) of SHR medulla (data not shown). Further studies are ongoing to investigate which nuclei and what types of neurons and glial cells are involved in the proteomic responses to acupuncture in SHRs.

We would like to state that this study does not provide direct evidence for the involvement of oxidative stress reduction in hypertension. Our results show a strong correlation between acupuncture treatments, upregulation of antioxidant enzymes, and reduction of hypertension. In the future, our laboratory will conduct long term studies to generate direct evidence of this involvement.

ALDH2, an enzyme involved in the activation of NO compounds, plays a role in the bioactivation of multiple drugs used for heart failure, such as glyceryl trinitrate and pentaerythritol tetranitrate. Although acupuncture does not directly lower blood pressure to normal level in SHRs, the resulting up-regulation of ALDH2 suggests that it could be used as a complementary treatment to modern medicine to increase the efficacy of certain nitric oxide based drugs. Additionally, hypertension has been shown to result in decreases of endothelial and neuronal nitric oxide synthase (eNOS & nNOS), while acupuncture has been shown to stop the reduction of nNOS and eNOS in SHRs [43], [44], [45]. The increased levels of nNOS as a result of hypertension directly affect NO levels, which is a potent vasodilator, further supporting our hypothesis that acupuncture modulates hypertension through multiple, simultaneously acting pathways.

The synapsins are a family of phosphoproteins present in neurotransmitter vesicles that are essential to normal neurotransmitter release at the synaptic cleft. Studies have shown that double knockout (synapsin-1 and synapsin-2) mice have a lower basal blood pressure than normal mice, and additional studies from the same group showed that the same double knockout mice did not respond with progressive hypertension to treatment with cyclosporine A as is normally seen in mice, indicating that the synapsins play an integral role in control of blood pressure [46]. As shown from our proteomic panel, synapsin-1 is significantly down-regulated in the Taichong group when compared to the model group. Down-regulation of synapsin could be another parallel avenue through which acupuncture helps control hypertension in SHRs.

Currently, there are no globally accepted biomarkers that readily differentiate between acupuncture at acupoints and acupuncture at a sham location. One of the biggest issues facing acupuncture research is the lack of a reliable method to determine if acupuncture was properly administered. Identification of reliable biomarker(s) for acupuncture would be a powerful tool for researchers to validate the efficacy of acupuncture treatments and optimize acupuncture regimen. Although the modulation of the medullar levels of oxidative stress enzymes cannot be used as a clinical biomarker for acupuncture, it may be useful for validation of acupuncture in animal studies. Adaptation of these changes in specific protein levels in the plasma, urine, or other easily accessible samples, is the next step in development and validation of these alterations as potential biomarkers for acupuncture. Other reports [37], [38] have corroborated our results on a much smaller scale, showing acupuncture does in fact up-regulate SOD and glutamate dehydrogenase, however, our complete proteomic expression panel is a more useful and accurate measure of successful acupuncture as the proteomic response is available for more proteins.

In this study, the Western blot analysis revealed that the expression of synapsin I remained unchanged after acupuncture treatment at non-acupoint but was down-regulated after acupuncture treatment at Taichong point (see Figure 5). In contrast, MBP remained unchanged after acupuncture treatment at non-acupoint but was up-regulated after acupuncture treatment at Taichong point. Western blot analysis can provide useful semi-quantitative data on protein expression. However, this commonly used assay can easily generate inconsistence of protein expression data given that most proteins are subject to degradation and modification by a number of factors.

In this study, we have observed some inconsistent expression data of individual proteins/genes when distinct techniques/assays are used. For example, although the results of MBP by Western blot assay were consistent with those by qRT-PCR, but were opposite to those from the 2-D gel analysis. Unlike Western blot and 2D gel assays, RT-PCR assays can generate quantitative data of gene expression at mRNA levels. Theoretically, the expression data of a specific gene at both protein and mRNA levels should be consistent. However, these data may be inconsistent due to the following reasons: a) mRNA is unstable and fragile; b) mRNA can be modified and thus its translation into the protein can be altered; and c) transcription and translation are regulated by a number of epigenetic and genetic factors and these two processes can be uncoupled under some conditions.

The clinical implication of our current study is unclear but may implicate the potential use of antioxidants in hypertension management. Potential sources of excessive ROS in hypertension include nicotinamide adenine dinucleotide phosphate (NADPH) oxidase, mitochondria, cyclooxygenase 1 and 2, cytochrome P450 epoxygenase, xanthine oxidase, endothelium-derived NO synthase, and transition metals [47]. A number of epidemiological and clinical data suggests that antioxidant-rich diets or natural antioxidants reduce blood pressure and cardiovascular risk [48], [49], and animal studies have also shown the antihypertensive activity of antioxidants [50], [51]. However, the majority of randomized clinical studies using natural antioxidants including include vitamins A, C and E, L-arginine, flavanoids, and mitochondria-targeted agents (coenzyme Q10, acetyl-L-carnitine, and α-lipoic acid have given disappointing or conflicting results [47]. Further mechanistic studies are needed to elucidate the role of ROS in the pathogenesis of hypertension and if proper use of antioxidants in combination of conventional antihypertensive agents benefits hypertensive patients.

Our current study focused on the “acute” effect over 1 week and identification of proteomic responses for the short-term effect of acupuncture on hypertension. From that point of view, we thought it to be less important to measure SBP before acupuncture each day, and focus more on the proteomic responses through which changes occur. We knew that to study the proteomic response to acupuncture, the rats would be sacrificed shortly after the experiment. Thus, it would be difficult to completely study the long term (e.g. >1 month) effect and sustainability of acupuncture on hypertension. In the future, we would be interested in performing studies to unfold the long-term effect of acupuncture on hypertension.

In conclusion, this study is the first to report the complete protein expression profile of acupuncture on SHRs, and has shown the potential involvement of oxidative stress as a partial mechanism in reduction of blood pressure by acupuncture therapy. Further studies are warranted to investigate the role of oxidative stress modulation by acupuncture in the treatment of hypertension.
